# Spatial and temporal evolution of Guangdong tourism economic network structure from the perspective of social networks

**DOI:** 10.1016/j.heliyon.2023.e18570

**Published:** 2023-07-21

**Authors:** Lijuan Zhang, Azizan Marzuki, Zhenjie Liao, Kaixiong Zhao, Zhihao Huang, Wenfu Chen

**Affiliations:** a School of Housing, Building and Planning, Universiti Sains Malaysia, Penang, Malaysia; b School of Management, Guangzhou Huashang College, Guangzhou, China; c School of Media and Communication, Guangzhou Huashang College, Guangzhou, China

**Keywords:** Tourism economy, Network space structure, Social network, Space–time evolution, Guangdong province

## Abstract

This study uses social network analysis and modified gravity model methods to empirically analyse the network spatial correlation structure and spatiotemporal development trend of 21 cities in Guangdong Province from 2000 to 2020 based on tourism economic development data. The findings show that, first, Zhuhai has the greatest potential for growth as the centre of the spatial and temporal evolution trend of the network structure of the tourism economy in Guangdong Province, ahead of Shenzhen, Huizhou, Zhaoqing, Zhongshan, Jiangmen and Dongguan. However, Guangzhou, the capital city of Guangdong Province, is experiencing a decline in such influence and development. Second, there is a counter-trend growth in the number of tourism-related economic links among the 21 cities. Although Guangdong's tourism economic network intensity is strong, there is still room for further optimisation. Third, the results of the overall network indicators show that there is a need for further improvement in network density, grade and efficiency to help reduce the relative development gap of the cities' tourism and effectively improve the overall development of Guangdong's tourism economy. Finally, based on the core–periphery structure, this study proposes relevant suggestions for the sustainable development of Guangdong's tourism industry.

## 1 Introduction

China's tourist economy has progressed significantly since the opening of its economy, thus attracting significant academic attention [1]. Many foreign scholars have examined tourism agglomeration, space–time differentiation, convenient transportation, the sustainability of the tourism economy, the protection of historical relics, carbon emissions and other issues. Most studies have focused on the construction of transportation infrastructure, coordinated development of the environment, cultural capital, network structure and rural revitalisation [2]. The complex tourism economic network system comprises various tourism elements in a region, reflecting the role of various links in and their impact on interregional tourism and regional economic behaviour [3]. The tourism economic network in the Yellow River Basin, the Guanzhong Plain, Southwest China, the middle reaches of the Yangtze River and the Guangdong–Hong Kong–Macao Bay Area has been widely explored [4]. Among them, several studies have examined provincial capital cities such as Yunnan, Inner Mongolia, Xinjiang and Wuhan; however, the spatiotemporal evolution of Guangdong's tourism economic network characteristics remains understudied [5].

The largest economic province in the country, Guangdong Province is a provincial administrative region of the People's Republic of China. In the past 20 years, Guangdong's tourism industry has entered a golden period of rapid development. Since 2000, some major indicators such as total tourism revenue, tourism foreign exchange income and cultural industry have added value to Guangdong Province, which has ranked first in China for several consecutive years [6]. The People's Government of Guangdong Province issued the ‘Implementation Plan for the ‘14th Five-Year’ Tourism Industry Development Plan of Guangdong Province’. The plan proposes that by 2025, significant progress will be made towards creating a robust tourism province and the tourism industry's main indicators will continue to be the best in China. However, as Guangdong Province's tourism economy has grown rapidly, there has been an obvious imbalance and diversity in the development level across the province.

As a major tourism province in China, there is a significant gap in the level of development across cities in Guangdong Province despite the increasingly growing tourism economic links. Therefore, as typical and representative, this study selects the Guangdong Province to promote the coordinated development of tourism and enhance regional tourism competitiveness. To better understand the trend of Guangdong's tourism development, we use social network analysis to study the space–time development of the region's tourism economy.

Social network analysis, originating from Western sociology in the 1930s, is a methodology to study the relationship between societies and their structural characteristics from the perspective of group mechanics based on graph theory [7]. It provides a theoretical paradigm and a research method suitable for analysing the network structure of complex systems. Since its appearance in the 1930s, Western sociology has accepted social network analysis due to its intuitive performance and accurate quantification. In the 21st century, the ‘fit’ between the complex and changeable tourism system and social network analysis has attracted the attention of tourism scholars, becoming a unique way to study tourism development and providing an excellent opportunity to study various relationships between complex tourism systems [8]. In recent years, many studies have focused on social network analysis in tourism [9,10]. The research mainly focuses on interorganisational relationships [11,12], virtual networks, scenic spots and destination networks [13,14]. Additionally, the depth and breadth of urban networks from the perspective of tourism are being explored [15]; however, these studies have mainly focused on the ranking of cities [16] and ignored the driving mechanisms of urban tourism economic networks [17,18]. The urban tourism network can be understood in more detail from the perspective of dynamics and evolution [19,20]. Currently, only two studies are related to the network structure of the tourism economy in Guangdong Province. One of these studies has focused mainly on 11 cities in the Guangdong–Hong Kong–Macao Greater Bay Area, including the Hong Kong Special Administrative Region, the Macao Special Administrative Region, Guangzhou, Shenzhen, Zhuhai, Foshan, Huizhou, Dongguan, Zhongshan, Jiangmen and Zhaoqing of Guangdong Province [21]. Furthermore, the study's time panel selects 2007, 2012 and 2017 to analyse the tourism economic linkages of these 11 cities instead of all cities of Guangdong Province. Therefore, the study findings are of no reference value. Guangdong Province is the subject area of another study that uses four periods of 2000, 2006, 2012 and 2018 to analyse the correlations of tourism economy among cities using the social network analysis method and examine the overall and individual network characteristics and their dynamic evolution and analyse the status of cities at different stages [22]. The study found the following points: first, the tourism economic correlation in Guangdong Province is increasing continuously, and higher correlation strength appears in areas in the Pearl River Delta region. Second, the network level has reduced, showing a core–periphery with a prominent centre and subsidence around the Pearl River Delta structure. Third, the overflow and benefit pattern of the tourism economy is unbalanced, with the overflow and benefit concentrated mainly in the Pearl River Delta cities represented by Guangzhou and Shenzhen. The overall regional imbalance has weakened, and there is still a need to optimise the spatial pattern of the tourism economy [23].

However, these studies still have some drawbacks. First, in terms of the research area, the research on the characteristics of the tourism economic network in a single province (Guangdong Province) is inadequate. Second, the time panel ended in 2018; the selected period is 6 years. There is a need for research covering longer time spans and intervals; furthermore, the impact of the COVID-19 pandemic on the tourism economy should be considered.

Accordingly, we use social network analysis to study the temporal and spatial characteristics and evolution trends of the network structure of Guangdong's tourism economy from 2000 to 2020. We also propose targeted development countermeasures to bridge the limitations of the current literature in this field. These countermeasures will help provide reference suggestions for the coordinated development of Guangdong's tourism economy and help improve the overall tourism competitiveness of Guangdong Province. These aspects ensure that the study has value and practical significance. This article expands the research on the network structure of tourism economy from the perspective of social networks, not only providing a useful supplement to existing literature, but also providing relevant suggestions for the sustainable development of Guangdong's tourism industry.

## 2 Study area, methods and data sources

### 2.1 Study area

This study focuses on Guangdong Province, which comprises 21 cities segregated into four regions: Pearl River Delta (Southern Guangdong), Eastern Guangdong, Western Guangdong and Northern Guangdong. The Pearl River Delta includes nine administrative cities: Guangzhou, Shenzhen, Zhuhai, Dongguan, Zhongshan, Foshan, Jiangmen, Huizhou and Zhaoqing. Guangzhou and Shenzhen are among the most developed Chinese cities. Eastern Guangdong includes four administrative cities: Shantou, Shanwei, Chaozhou and Jieyang. Western Guangdong includes three administrative cities: Zhanjiang, Maoming and Yangjiang. Northern Guangdong includes five administrative cities: Shaoguan, Heyuan, Meizhou, Qingyuan and Yunfu. Guangdong has abundant cultural and tourism resources, prominent industrial clusters, a high degree of marketisation, a relatively sound public service system and a sound foundation and conditions for the integrated development of culture and tourism. In 2020, Guangdong Province received 231 million overnight tourists (226 million domestic and 4.7 million overseas, including 804,600 foreigners and 2.8 million Hong Kong citizens). A total of 587,100 people came from Macau and 470,900 from Taiwan. Guangdong Province's total tourism revenue in 2020 was 469.1 billion yuan, of which US$2.3 billion was foreign exchange earnings from international tourism and 452.8 billion yuan was domestic tourism revenue (from the 2020 Guangdong Province National Economic and Social Development Statistical Bulletin).

### 2.2 Methods

The British scholar Brown first proposed the concept of social network [24], which was later adopted and widely used in many fields, including innovation, urbanisation, tourism and economy. Social network analysis was used in this study because it is considered a strong paradigm for spatial research in tourism [25]. This research method can help to understand the spatial correlation of the tourism economy, examine the structural characteristics and temporal and spatial evolution trends of the tourism economic network, analyse the status and role of each city and understand the current situation of the tourism economy in Guangdong Province.

#### 2.2.1 Gravity model of tourism economic linkage

Gravity models in economics are derived from the law of universal gravitation [26]. Universal gravitation describes the interaction and mutual influence relationships between objects. Taaffe proposed that the strength of economic ties is proportional to the product of the population between cities and inversely proportional to the square of the distance between cities [27]. Since then, the gravity model has been widely used to study regional economic interactions [28]. The gravitational model means that there is an attractive force between objects, and the strength of the gravitational force is proportional to the mass (scale) of the two objects and inversely proportional to the square of the distance between the two objects. Scholars generally use the factors of total tourism revenue, the number of tourists and the shortest road distance between cities to reflect the strength of regional tourism economic correlation [29]. However, as cities of different sizes and grades contribute differently to the tourism economic correlation between two cities, the tourism economic correlation should be directional and the gravity model should be corrected according to the different contributions of the two cities.

The total GDP is the most direct factor for measuring a city. Therefore, based on the original gravity model, this study adds the factor of total GDP, expresses the ‘quality’ of the tourism economy by the total number of tourists, total tourism income and total GDP and represents the ‘scale’ by the total number of tourists × total tourism income × total GDP data. The ‘shortest road distance’ is adopted as the ‘distance’ data. The ratio of the total GDP to the sum of the total GDP of the two cities is used as the correction coefficient to conform to the directional characteristics of the attractiveness of the tourism economy between cities [30].

(1) $$Y_{ij}=K_{ij}\frac{\sqrt[{3}]{P_{i}T_{i}G_{i}}\sqrt[{3}]{P_{j}T_{j}G_{j}}}{{D_{ij}}^{2}},K_{ij}=\frac{G_{i}}{G_{i}+G_{j}}$$

where *Kij* is the gravitational coefficient and *Yij* is the tourism economic linkage intensity between two cities. *Pi* and *Pj* are the number of tourists in the year, *Ti* and *Tj* are the tourism income of the two cities in the year, *Gi* and *Gj* are the total GDP of the two cities in the year and *Dij* is the distance between two cities.

#### 2.2.2 Overall network characteristic indicators

This study uses the social network analysis method to analyse the overall network spatial correlation characteristics of the tourism economic development of Guangdong Province comprising 21 cities by calculating the values of network density, correlation degree, grade degree and efficiency.

The network density is the ratio between the number of own relationships and the maximum possible in the entire network. Its value reflects the density of tourism economic relationships between cities [31].

(2) $$D=\frac{L}{N\times \left(N-1\right)}$$

where *D* is the network density, *L* is the number of actual relationships and *N* is the number of regional cities.

Network connectedness reflects the robustness and vulnerability of the tourism economic development cyberspace association itself. When many lines in the urban tourism economic development cyberspace association pass through a certain point (city), the network has a lower degree of correlation and therefore weaker robustness. In contrast, when the spatial correlation network line is not distributed around a single point, the network will have a greater correlation, making it more robust [32].

(3) $$C=1-\frac{V}{N\times \left(N-1\right)/2}$$

where *C* is the correlation degree, *V* is the logarithm of unreachable points in the network and *N* is the number of regional cities.

Network hierarchy measures the degree of asymmetric arrival between cities in the network. The higher and stricter the network level, the more dependent and marginal the cities are in the network space structure [33].

(4) $$H=1-\frac{K}{\max\left(K\right)}$$

where *H* is the degree of hierarchy, *K* is the logarithm of symmetrically reachable points in the network and max (*K*) is the logarithm of the maximum possible reachable point.

Network efficiency reflects the efficiency of connections between cities in the cyberspace connection of tourism economic development. The lower the network efficiency, the more the strengthening of connections and the reinforcing of tourism economic links needed between cities [34]. A stabler spatial connection network for tourism economic development makes it easier to promote the development of the economy through the spatial connection network [35].

(5) $$E=1-\frac{M}{\max\left(M\right)}$$

where *E* is the network efficiency, *M* is the number of redundant lines in the network and max (*M*) is the maximum possible number of redundant lines.

#### 2.2.3 Individual network characteristic indicators

The analysis of individual network structure features mainly adopts the centrality of the social network midpoint analysis [36]. Point centrality analysis includes the centrality of the point and its middle and near centrality [37]. Pointwise centrality measures the degree of contact between two participants [38]. The higher the centrality of the point, the closer the actor is to other actors and the greater the actor's power. In a directed graph, the pointwise centrality is divided into ‘point-in degree’ and ‘point-out degree’. Intermediate centrality measures the ability of participants to control other participants [39]. If a point is located on the connection path of the other two points, it has a high intermediate centrality, meaning that the point plays the role of a communication bridge in the connection [40]. Near centrality is the opposite of middle centrality. It measures the ease of contact between participants, i.e. the ability to resist being controlled by other participants. The closer the centrality the less controlled the point. The calculation formula for each centrality is as follows [41]:

CAD = Degree of point.

CRD=(Point penetration + Point out)/(2n-2)

(6) $$C_{ABi}=\sum_{j}^{n}\sum_{k}^{n}b_{jk},j\neq k\neq i,j<k$$

(7) $$C_{RBi}=\frac{2C_{AB}}{n^{2}-3n+2}$$

$C_{APi}^{-1}=\sum_{j=1}^{n}d_{ij}$,*d*_*ij*_ is the shortcut path between points i and j.

(8) $$C_{RPi}^{-1}=\frac{C_{APi}^{-1}}{n-1}$$

where $C_{AD}$ is the absolute degree centrality of a point, $C_{RD}$ is the relative degree centrality of a point and *n* is the scale of the network. $C_{ABi}$ is the absolute middle centrality of point i and $C_{RBi}$ is the relative middle centrality of point *i*. $C_{APi}^{-1}$ is the absolute approach centrality of point *i* and $C_{RPi}^{-1}$ is the relative approach centrality of point *i*.

#### 2.2.4 Core edge model

We adopt core edge analysis to scientifically and extensively examine its internal spatial structure [42,43]. Using the Ucinet6 software, this model can be employed to show the position of each city node clearly and intuitively in the tourism economic network, indicate the spatial characteristics of the tourism economic network of 21 cities in Guangdong Province and provide relevant development countermeasures [44,45].

### 2.3 Data source

The dataset consists of panel data from 2000 to 2020 for the number of tourists (1000 persons), tourism income (100 million yuan), gross domestic product (100 million yuan) and per capita gross domestic product (100 million yuan) in 21 cities in Guangdong Province. The above data are obtained from the statistical bulletins concerning the national economic and social development of each city in the corresponding year, the China Tourism Statistical Yearbook, the websites of the Guangdong provincial and municipal tourism bureaus, the websites of the National Bureau of Statistics and the China Statistical Yearbook. Considering the difficulty and representativeness of data acquisition, the years 2000, 2005, 2010, 2015 and 2020 are selected as time sections, with time nodes evenly selected every 5 years.

## 3 Results

### 3.1 Overall network structure indicators of the cities

**Fig. 1:**
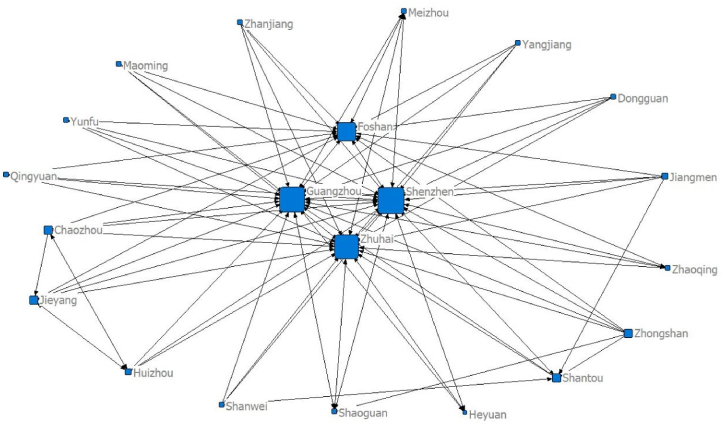
The overall network structure of Guangdong's tourism economic development in 2000 Source: Statical yearbook of China and own calculations.

**Fig. 2:**
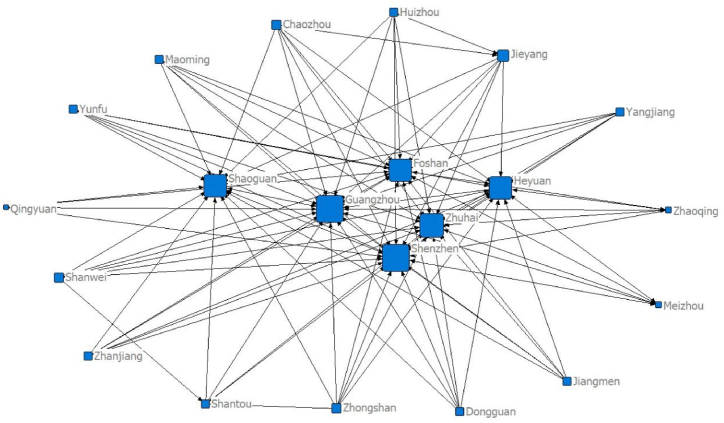
Overall Network Association Structure of Guangdong Tourism Economic Development in 2005 Source: Statical yearbook of China and own calculations.

**Fig. 3:**
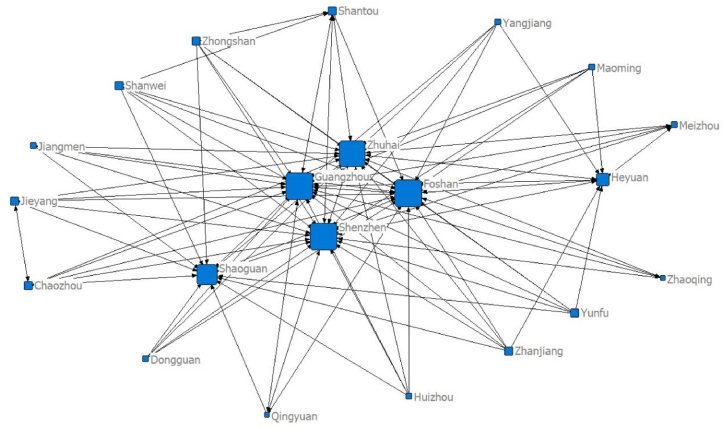
The overall network structure of Guangdong's tourism economic development in 2010 Source: Statical yearbook of China and own calculations.

**Fig. 4:**
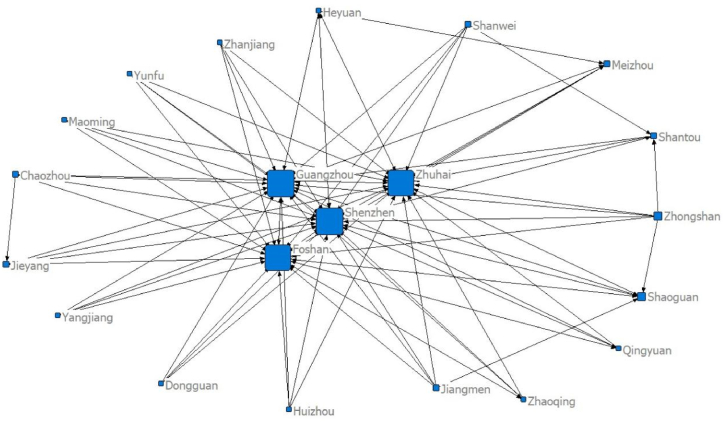
The overall network structure of Guangdong's tourism economic development in 2015 Source: Statical yearbook of China and own calculations.

**Fig. 5:**
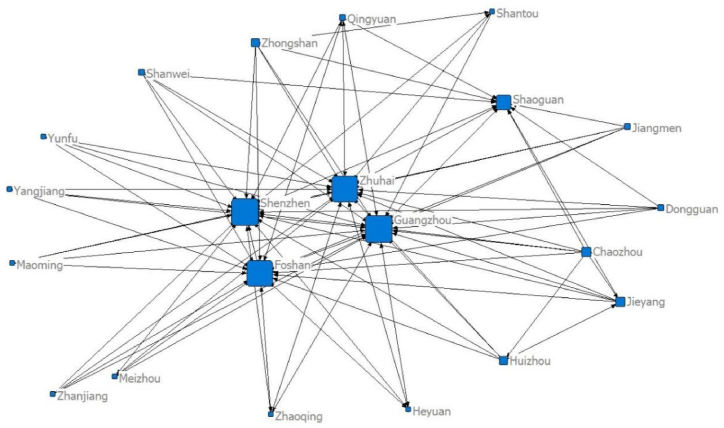
The overall network structure of Guangdong's tourism economic development in 2020 Source: Statical yearbook of China and own calculations.

Using the panel data of 21 cities in Guangdong Province and the modified gravity model, the spatial relationship matrix of tourism economic development between cities is constructed. Netdraw, the visualisation tool of the Ucinet software, is used to draw the data for five separate time points of the network structure: 2000 (92 relationships), 2005 (114 relationships), 2010 (106 relationships), 2015 (91 relationships) and 2020 (97 relationships), as seen in Fig. 1, Fig. 2, Fig. 3, Fig. 4, Fig. 5, respectively. Fig. 1, Fig. 2, Fig. 3, Fig. 4, Fig. 5 show that the overall spatial connection network structure of the Guangdong tourism economy is obvious, with Guangzhou, Shenzhen, Zhuhai and Foshan at the core of the network. In 2005 and 2010, based on the original network core of Guangzhou, Shenzhen, Zhuhai and Foshan, Shaoguan and Heyuan were integrated into the network core, with the shape of ‘one core and two wings’. In 2015 and 2020, Shaoguan and Heyuan migrated from the core network centre circle. The reasons for the above changes lie in the following point: Guangzhou, Shenzhen and Foshan have strong tourism potential.

The Pearl River Delta region has a relatively high intensity of tourism economic correlation and total gravitational force of each city. It is the hinterland of the tourism economy in Guangdong Province. This conclusion is incontestable for the following reasons: first, Guangzhou is the capital city of Guangdong Province and is certainly impacted by factors such as transportation and economy. Second, Foshan is spatially closer to Guangzhou, offering convenient transportation and rich tourism resources. Third, Shenzhen and Zhuhai are the second and third largest cities in Guangdong Province. Fourth, in 2017, the Guangdong–Hong Kong–Macao Greater Bay Area policy was proposed. The nine cities (Guangzhou, Shenzhen, Zhuhai, Dongguan, Zhongshan, Foshan, Jiangmen, Huizhou and Zhaoqing) in the Pearl River Delta of Guangdong Province are all a part of the Bay Area. The rapid development of regional integration in the Pearl River Delta has improved its interaction with Shaoguan and Heyuan, two major cities in Northern Guangdong. In 2005, the tourism economic network of 21 cities in Guangdong Province expanded significantly (from 92 to 114). The tourism economic network intensity decreased between 2010 and 2015, reaching the lowest level in history in 2015, before marginally improving in 2020.

#### 3.1.1 Network density

Using the above formula, the tourism economic spatial network density of Guangdong Province in 2000, 2005, 2010, 2015 and 2020 is calculated along the network/density path in the Ucinet6 software. Table 1 shows that the tourism economic network density of the province in 2000, 2005, 2010, 2015 and 2020 was 0.219, 0.271, 0.222, 0.217 and 0.231, respectively. The network density is always lower than 0.5, indicating that the tourism economic ties in Guangdong Province are weak with a big potential for improvement, thus cooperation should be strengthened. In 2015, the tourism economic network density of Guangdong Province reached the lowest level in history. In 2020, the tourism economic ties rose by 64.5%, meaning that after the outbreak of the COVID-19 pandemic, the tourism economic network showed a counter-trend growth in 2020.

**Table 1:** Tourism economic network density of 21 cities in guangdong province from 2000 to 2020.

years	Density	Number of ties	Standard deviation	Average Density
2000	0.219	92	0.414	4.381
2005	0.271	114	0.445	5.429
2010	0.252	106	0.434	5.048
2015	0.217	91	0.412	4.333
2020	0.231	97	0.421	4.619

#### 3.1.2 Network relevance, network grade and network efficiency

Using the above formula and following the network/connectedness path in the Ucinet6 software, we find that the spatial network correlation degree of the tourism economy in the Guangdong Province in 2000, 2005, 2010, 2015 and 2020 is 1. This shows that the spatial network of the tourism economy in the region is not developed only around one city. Instead, the economy is well developed, and the network has a clear spatial correlation and spillover effects.

**Fig. 6:**
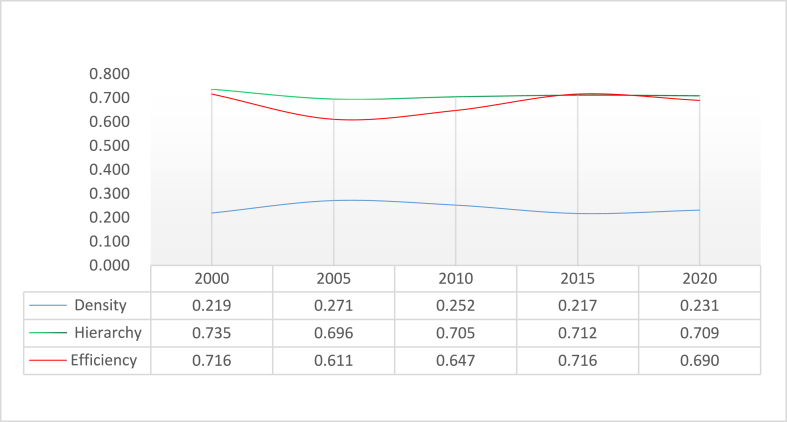
Summary of related network structure indicators of tourism economic development in Guangdong province from 2000 to 2020.

Cities have both direct and indirect tourism economic relationships, and the network structure is stable. Fig. 6 shows the changing trend of network grade and efficiency. The calculation results of network grade show an overall but insignificant trend of decline from 2000 to 2020. It was 0.7347 in 2000 and 0.7089 in 2020, showing a decrease of 35.1%. Fig. 6 shows that the degree of the tourism economic network in the Guangdong Province remains high above 0.5 with obvious hierarchical characteristics, and the network structure should be further optimised. The overall network efficiency shows a downward trend with slight fluctuations in some years. The tourism efficiency in 2020 has increased compared with 2005 and 2010 and decreased compared with the highest value in 2015 (the value is the same as that in 2000). The connection of tourism economic development among cities in Guangdong Province is increasing, the stability of the network is gradually improving and the cities participating in the coordination and cooperation of tourism development in the region are developing.

### 3.2 Analysis of individual network structure indicators of 21 cities in Guangdong Province

With the help of (6), (7), (8), the point-to-point centrality, the proximity centrality and the intermediary centrality of the 21 cities in Guangdong Province from 2000 to 2020 are calculated along the network/centrality path using the Ucinet6 software.

#### 3.2.1 Analysis of pointedness and centrality

**Fig. 7:**
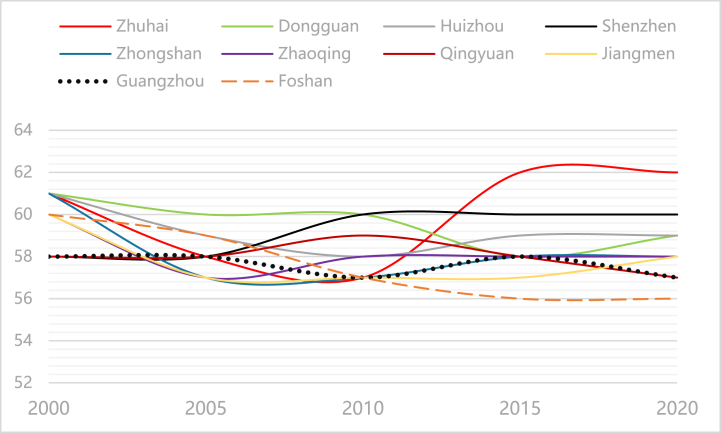
Change Trend of Top 10 Cities in Guangdong Province from 2000 to 2020 Source: Statical yearbook of China and own calculations.

Table 2 shows that the average point centrality of the 21 cities in Guangdong Province is 56.4, with 10 cities having above-average values, namely, Zhuhai (60), Dongguan (59.6), Shenzhen (59.2), Huizhou (59.2), Zhongshan (58.2), Zhaoqing (58.2), Qingyuan (58), Jiangmen (57.8), Guangzhou (57.6) and Foshan (57.6). Four cities (Zhuhai, Dongguan, Shenzhen and Huizhou) have values higher than 59, which shows that they have a high position in the spatial connection network of tourism economic development in the province. Among them, from 2000 to 2020, the influence of the tourism spatial connection network centre has grown significantly and rapidly in Zhuhai, showing a decline from 2000 to 2010—a steep rise after 2010 and a relatively stable feature. From 2000 to 2020, the cities showing a decline—a slight increase—tend to be stable. These include Shenzhen, Huizhou, Zhaoqing and Zhongshan. Jiangmen and Dongguan show a decline in growth, while the cities with decreased overall centrality and influence include Foshan, Guangzhou and Qingyuan. The trend route map shows that the city of Zhuhai has the greatest influence and development potential in the future tourism economic network, followed by Shenzhen, Huizhou, Zhaoqing, Zhongshan, Jiangmen and Dongguan. Foshan demonstrated the highest influence of the tourism economic network, while Guangzhou and Qingyuan showed a declining influence (see Fig. 7).

**Table 2:** Centrality of Guangdong Province from 2000 to 2020.

City	2000	2005	2010	2015	2020	Mean value	Sort
Guangzhou	58	58	57	58	57	58	7
Shenzhen	58	58	60	60	60	59	3
Zhuhai	61	58	57	62	62	60	1
Shantou	53	50	55	56	53	53	13
Foshan	60	59	57	56	56	58	7
Shaoguan	58	54	55	56	55	56	9
Heyuan	56	53	54	54	54	54	12
Meizhou	57	54	55	55	55	55	10
Huizhou	61	59	58	59	59	59	3
Shanwei	58	53	54	55	55	55	11
Dongguan	61	60	60	58	59	60	2
Zhongshan	61	57	57	58	58	58	4
Jiangmen	60	57	57	57	58	58	6
Yangjiang	58	54	55	56	56	56	8
Zhanjiang	58	54	54	56	56	56	9
Maoming	58	54	55	56	56	56	8
Zhaoqing	60	57	58	58	58	58	4
Qingyuan	58	58	59	58	57	58	5
Chaozhou	52	49	52	53	49	51	15
Jieyang	53	48	52	56	52	52	14
Yunfu	58	54	54	56	56	56	9
Minimum value	52	48	52	53	49	51	/
Maximum value	61	60	60	62	62	61	/
Average value	58	55	56	57	56	56	/
Standard deviation	3	3	2	2	3	3	/

**Fig. 8:**
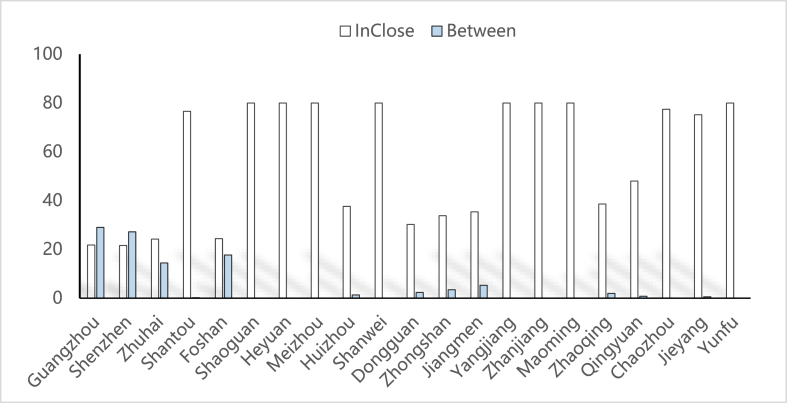
Comparison of point in degree and point out degree Source: Statical yearbook of China and own calculations.

In the tourism economic network structure of the 21 analyzed cities, five cities led in terms of highest penetration rate: Heyuan (6.2), Chaozhou (6.2), Jieyang (5.8), Shanwei (5.4) and Guangzhou (5.4) (Table 3). The high point-out performance includes Shenzhen (18.4), Guangzhou (18.2), Zhuhai (16.2), Foshan (15.6), Dongguan (9.8), Zhongshan (6.2), Jiangmen (4.6) and Huizhou (3.6) (Table 4). Fig. 8 illustrates those six cities—Guangzhou, Shenzhen, Zhuhai, Foshan, Dongguan and Zhongshan—having a high degree of point entry and exit. These cities show a high willingness to travel and have a strong attraction for tourists from other cities. The tourism economy is quite dynamic. Simultaneously, the average point-out degree of these six cities is far higher than the average point-in degree, indicating that the willingness to travel is greater than their own tourism attraction. This is because these nine cities are located in the Pearl River Delta region of Guangdong Province and their economies are more developed than those of southern, eastern and Western Guangdong. At the same time, these cities have unique transport advantages and a rich array of urban attractions, indicating greater attractiveness for tourism. The per capita GDP of these cities is higher than that of others. Residents of these cities have greater willingness and ability to travel. Shantou, Zhaoqing, Qingyuan, Chaozhou and Jieyang are cities with higher point entry than exit. In Huizhou and Jiangmen, the point-in and point-out degrees are similar. However, Shaoguan, Heyuan, Meizhou, Shanwei, Yangjiang, Zhanjiang, Maoming and Yunfu have only a point entry degree but no point exit, which shows that tourism resources are highly attractive, the willingness to travel is low and they are not active in the tourism economic network structure of Guangdong Province as a whole. In terms of their tourist attractions, Heyuan (6.2), Chaozhou (6.2), Jieyang (5.8), Shanwei (5.4) and Guangzhou (5.4) ranked higher than Shenzhen, Zhuhai, Foshan, Dongguan and Zhongshan.

**Table 3:** Entry and mean value of each time node in Guangdong Province from 2000 to 2020.

Node	2000	2005	2010	2015	2020	Mean value
Guangzhou	6	5	5	5	6	5
Shenzhen	6	5	4	4	4	5
Zhuhai	4	5	6	3	3	4
Shantou	5	6	5	4	5	5
Foshan	4	4	5	6	6	5
Shaoguan	4	6	5	4	5	5
Heyuan	6	7	6	6	6	6
Meizhou	5	6	5	5	5	5
Huizhou	3	3	4	3	3	3
Shanwei	4	7	6	5	5	5
Dongguan	3	2	3	4	3	3
Zhongshan	3	5	5	4	4	4
Jiangmen	4	5	5	5	4	5
Yangjiang	4	6	5	4	4	5
Zhanjiang	4	6	6	4	4	5
Maoming	4	6	5	4	4	5
Zhaoqing	4	5	4	4	4	4
Qingyuan	4	4	4	4	5	4
Chaozhou	6	7	6	5	7	6
Jieyang	5	8	6	4	6	6
Yunfu	4	6	6	4	4	5

**Table 4:** Point output and mean value of Guangdong Province from 2000 to 2020.

Node	2000	2005	2010	2015	2020	Mean value
Guangzhou	19	18	18	18	18	18
Shenzhen	20	18	18	18	18	18
Zhuhai	17	15	15	17	17	16
Shantou	2	1	0	0	2	1
Foshan	14	15	17	16	16	16
Shaoguan	0	0	0	0	0	0
Heyuan	0	0	0	0	0	0
Meizhou	0	0	0	0	0	0
Huizhou	4	4	5	3	2	4
Shanwei	0	0	0	0	0	0
Dongguan	4	15	14	6	10	10
Zhongshan	3	14	7	3	4	6
Jiangmen	4	5	5	5	4	5
Yangjiang	0	0	0	0	0	0
Zhanjiang	0	0	0	0	0	0
Maoming	0	0	0	0	0	0
Zhaoqing	2	4	3	2	2	3
Qingyuan	0	2	2	2	2	2
Chaozhou	1	1	1	0	0	1
Jieyang	2	2	1	1	2	2
Yunfu	0	0	0	0	0	0

#### 3.2.2 Analysis of intermediate centrality

The intermediate centrality index measures how far a city is located between two other cities and is an intermediate coordination and leading index. In the tourism economic network structure, cities with high middle centrality usually have more inbound and outbound tourists. The findings in Table 5 reveal a large difference in the index values of the middle centrality of the 21 cities in Guangdong Province. The centrality index of Guangzhou is 28.996, indicating a high control over other cities and a dominant position. Shenzhen ranked second, followed by Foshan and Zhuhai. The cities with the highest intermediate centrality are Guangzhou, Shenzhen, Foshan, Zhuhai, Jiangmen, Zhongshan and Dongguan. These cities have high tourism attractions and travel consumption. As intermediate cities connected by the tourism economic networking structure, they have strong control over node cities because of factors that are closely related to tourism resources, traffic location, consumption capacity and other factors. The middle centrality index of other cities is generally low, indicating that Guangdong is in a marginal position in the entire tourism economic network structure and is highly controlled by the central cities.

**Table 5:** Guangdong Province 2000–2020 intermediate centrality.

Node	2000	2005	2010	2015	2020	Mean value
Guangzhou	30	25	27	30	34	29
Shenzhen	35	21	17	33	30	27
Zhuhai	15	13	18	15	11	14
Shantou	1	0	0	0	0	0
Foshan	9	13	21	22	23	18
Shaoguan	0	0	0	0	0	0
Heyuan	0	0	0	0	0	0
Meizhou	0	0	0	0	0	0
Huizhou	0	1	3	1	1	1
Shanwei	0	0	0	0	0	0
Dongguan	1	1	2	5	3	2
Zhongshan	1	6	3	2	5	3
Jiangmen	2	6	6	7	5	5
Yangjiang	0	0	0	0	0	0
Zhanjiang	0	0	0	0	0	0
Maoming	0	0	0	0	0	0
Zhaoqing	1	5	3	1	1	2
Qingyuan	0	1	1	1	1	1
Chaozhou	0	0	0	0	0	0
Jieyang	0	2	0	0	0	1
Yunfu	0	0	0	0	0	0
Minimum value	0	0	0	0	0	0
Maximum value	35	25	27	33	34	31
Average value	5	5	5	6	5	5
Standard deviation	10	7	8	10	10	9

#### 3.2.3 Proximity centrality analysis

The proximity centrality is opposite to the middle centrality. It measures the convenience of a city's connection with other cities, i.e. the ability of not being under their control. The higher the proximity to the centre, the less controlled by others. Shaoguan, Heyuan, Meizhou, Shanwei, Yangjiang, Zhanjiang, Maoming, Yunfu, Chaozhou, Shantou and Jieyang are near the top of the centrality index (see Table 6). A comparative analysis of the middle centrality index and the near neutrality index of the 21 cities of the Guangdong Province shows that the tourism economic network of these 11 cities is relatively scattered, with no strong participation in the tourism economic network structure.

**Table 6:** Centrality of Guangdong Province from 2000 to 2020.

Node	2000	2005	2010	2015	2020	Mean value
Guangzhou	21	22	22	22	22	22
Shenzhen	20	22	22	22	22	22
Zhuhai	23	25	27	23	23	24
Shantou	74	75	80	80	74	77
Foshan	26	25	23	24	24	24
Shaoguan	80	80	80	80	80	80
Heyuan	80	80	80	80	80	80
Meizhou	80	80	80	80	80	80
Huizhou	36	38	35	39	40	38
Shanwei	80	80	80	80	80	80
Dongguan	36	25	26	34	30	30
Zhongshan	37	26	33	37	36	34
Jiangmen	36	35	35	35	36	35
Yangjiang	80	80	80	80	80	80
Zhanjiang	80	80	80	80	80	80
Maoming	80	80	80	80	80	80
Zhaoqing	39	36	38	40	40	39
Qingyuan	80	40	40	40	40	48
Chaozhou	75	75	77	80	80	77
Jieyang	74	74	77	77	74	75
Yunfu	80	80	80	80	80	80
Minimum value	20	22	22	22	22	22
Maximum value	80	80	80	80	80	80
Average value	58	55	56	57	56	56
Standard deviation	24	25	25	25	24	25

**Fig. 9:**
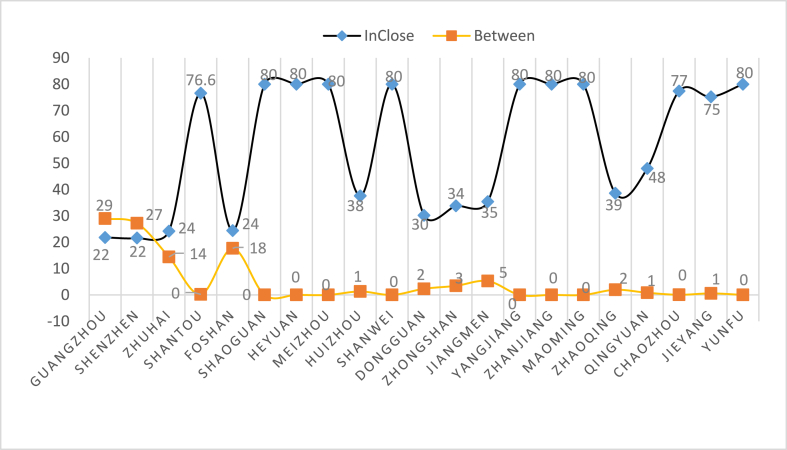
Comparison of the index of middle centrality and proximity centrality of 21 cities in Guangdong Province from 2000 to 2020 Source: Statical yearbook of China and own calculations.

By comparing the intermediate centrality index with the proximity centrality index (see Fig. 9), we find that for Guangzhou and Shenzhen, the intermediate index is higher than the proximity centrality index. The latter, however, is the lowest among many cities, which shows that at the core of the network of Guangdong's tourism economy, Guangzhou and Shenzhen have the highest power and influence in the tourism economic network structure of the region and can most conveniently communicate with other cities. Zhuhai has developed recently, showing that the near centrality index was higher than the middle centrality index. Foshan is a special case showing a more unique index image: the separation space between the index curve of proximity to centrality and intermediate centrality is the smallest, followed by that for Huizhou, Dongguan, Zhongshan, Jiangmen, Yangjiang, Zhaoqing and Qingyuan. These cities have low centrality in the middle (weak influence) and low proximity to centrality, indicating other cities have a significant impact on them. In addition, Shaoguan, Heyuan, Meizhou, Shanwei, Yangjiang, Zhanjiang, Maoming, Chaozhou, Jieyang and Yunfu have a high index of proximity to centrality and a low index of intermediate centrality, making them the weakest in the tourism economic network of Guangdong Province.

### 3.3 Analysis of the core–periphery model of the Guangdong tourism economic network structure

**Fig. 10:**
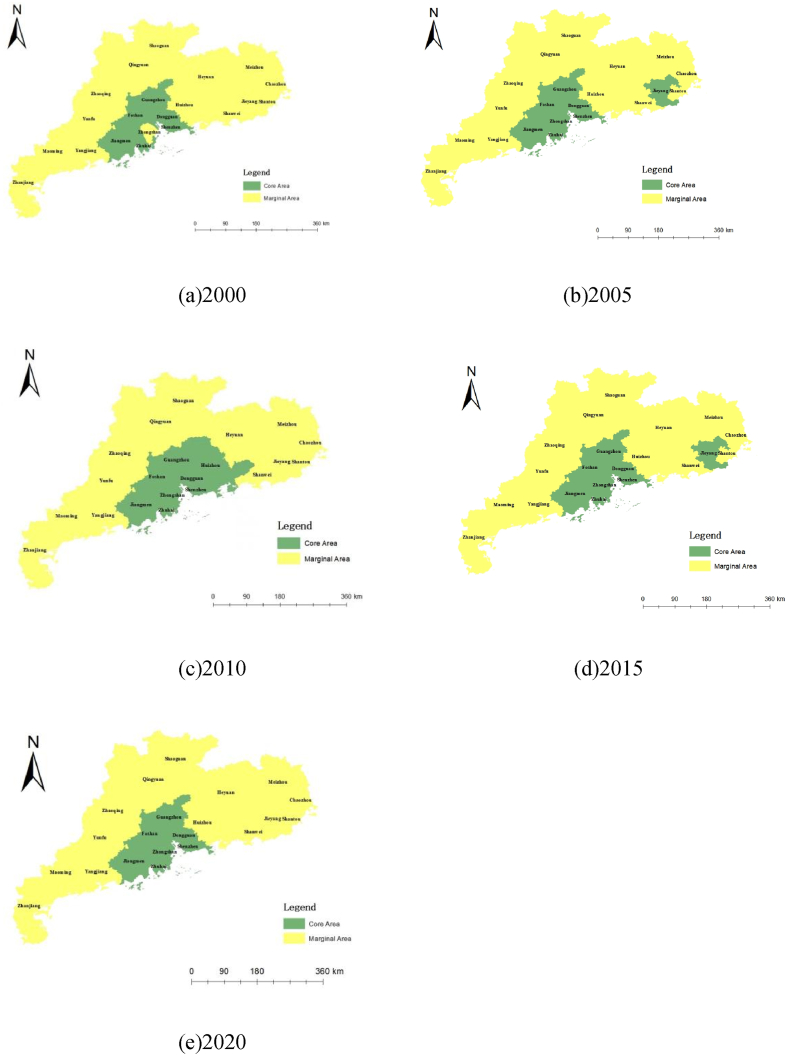
Structure Chart of Guangdong Tourism Economic Network from 2000 to 2020 (a) 2000; (b) 2005; (c) 2010; (d) 2015; (e) 2020 Source: Statical yearbook of China and own calculations.

The core–periphery programme in Ucinet6 software is used to analyse the core edge structure of the Guangdong tourism economic network space. The distribution results are shown in Fig. 10 (a), (b), (c), (d) and (e), which indicate that in the tourism economic network structure of the Guangdong Province, from 2000 to 2020, five nodes have become members of the core area: Guangzhou, Shenzhen, Zhuhai, Foshan and Dongguan, with four other nodes moving in the structure of the core and edge areas, namely, Zhongshan, Jiangmen, Huizhou and Jieyang. The other 12 nodes are stable at the edge structure.

**Fig. 11:**
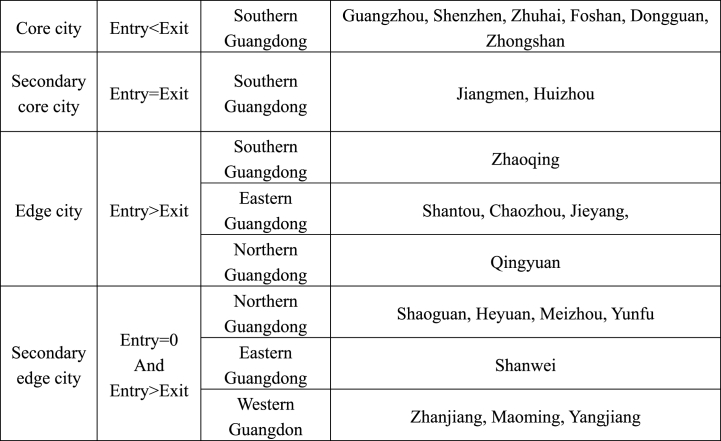
Four levels and secondary structure of the core edge of Guangdong's tourism economy.

By comparing and analysing the data presented in Figs. 1–5, 7 and 8 using a detailed comparison of the index values and the results of the point-degree centrality, point-out degree, point-in degree, proximity centrality and betweenness centrality of each city (Fig. 7, Fig. 8, Fig. 9) using intuitive pictures in Figs. 1–5 and 10, we have added four levels to the original core edge two-level structure as follows (Fig. 11).

## 4 Discussion

Compared with the existing research, this study has the following advantages. First, based on the original gravitational model, this study added the factor of total GDP, expressing the ‘quality’ of the tourism economy as the total number of tourists, total tourism revenue and total GDP, making the research data and conclusions more rigorous. Second, this study selects panel data of up to 21 years from 2000 to 2020 and evenly selects time nodes every 5 years, addressing the lack of original research in the time dimension. Third, this research identifies and presents the status and role of each city in the tourism economic network structure of Guangdong Province with fine-grained city-level data, specifically for the future of several important cities in the Pearl River Delta. Forecasting the changing trend, there is need for in-depth research on the characteristics of spatiotemporal evolution and development trends. Fourth, we added four levels to the original core edge two-level structure.

The shortcomings of this study are as follows. First, we use a modified gravity model to determine the spatial correlation of the tourism economy in Guangdong Province. In the future, spatial autocorrelation analysis and vector autoregressive model can be used to establish spatial correlation and compare the results to obtain complete advantages of different methods. Second, owing to time and space limitations, this study did not identify the factors and degrees that affect the tourism economy network structure in Guangdong Province; therefore, some data conclusions have not been explained in depth.

## 5 Conclusion

Based on the data on tourism economic development, this study analyses the characteristics and development of the spatial correlation structure of the tourism economy in Guangdong Province by adopting the social network analysis method and Ucinet6 software. The main conclusions are as follows.

First, the tourism economic network of the 21 prefecture-level cities in Guangdong Province has been gradually strengthened, showing a fluctuating upward trend. In general, the network layout structure of the Pearl River Delta is dense around the region. From 2000 to 2020, the province has seen a weak network density, with values lower than 0.5, indicating the need for further improvement and strengthened cooperation. In 2015, the tourism economic network density of Guangdong Province reached the lowest level in history. In 2020, however, the tourism economic ties indicated a growth rate of 64.5%, thus showing a counter-trend growth after the pandemic outbreak in 2020. The tourism economic network in Guangdong Province is maintained at a level above 0.5, which indicates obvious level characteristics, and the network structure should be further optimised.

Second, Guangdong's tourism economic network structure has a clear core and limitations. The core area is the Pearl River Delta (except Zhaoqing), followed by Jieyang in eastern Guangdong. Obvious differences exist in tourism development among regions in Guangdong Province; however, in recent years, these differences have gradually decreased.

Third, obvious differences exist in the active degree of tourism economic network structure among different regions in the province. The Pearl River Delta region is more willing to travel, even higher than its own tourism attraction, and its tourism economy is developing more actively. However, Guangdong's eastern, western and northern regions are more attractive, with lower willingness to travel; thus, they are not active in the overall tourism economic network structure.

Lastly, in the spatial connection network, Zhuhai has the greatest influence and development potential in the future tourism economic network, followed by Shenzhen, Huizhou, Zhaoqing, Zhongshan, Jiangmen and Dongguan. The influence of the tourism economic network of Foshan, Guangzhou and Qingyuan is declining.

From the node centrality (point, proximity and intermediary centrality), the spatial distribution characteristics and role positioning of the tourism economy in various regions of Guangdong Province are explored in detail, and the following development countermeasures are proposed.

First, focusing on the regional connotation and consolidating the characteristic industries is necessary. The economy in the region has developed rapidly, and various elements between and within regions have been continuously linked, developed and evolved. The distribution of administrative regions, the degree of planning tendency, the feelings about tourism and the popularity of tourist destinations differ greatly. Each region should focus on its regional characteristics, focus on its strengths and combine ‘culture + industry’ to further promote the long-term connotation and practical benefits. Tourism culture based on local characteristics and the traditional characteristics of Guangdong, such as the cultural Han Opera and the Chaoshan Kung Fu Tea, will be injected into the brand connotation to form an industrial chain, and clusters will provide practical benefits. The culture of Guangfu, Hakka, Chaoshan and Leizhou will continue to be inherited and carried forward. Further, it is essential to develop the online promotion of tourist destinations in Guangdong to promote regional culture and create multiple benefits in addition to economic benefits.

Second, the radiation role of central cities should be fully utilised, and adhering to the mode of developing multicore tourism in Guangdong Province is necessary. Furthermore, it is crucial to strengthen the radiation and driving role of Shenzhen, Guangzhou, Foshan, Dongguan and other cities and continue to improve Guangzhou's leading position as the tourism economic network of the region.

Moreover, there is a need to develop the role of Shenzhen, Guangzhou, Zhuhai and Foshan as the province's tourist distribution centres in transferring tourists to the surrounding cities and counties. The impact of one trillion cities, such as Shenzhen, Guangzhou, Foshan and Dongguan, on the tourism economy of Guangdong's marginal cities should be strengthened, which is not limited to developing their own tourism. For example, the focus should be on holiday short-distance leisure tourism in conjunction with surrounding cities and counties; the development of intercity transportation and tourism routes; the enhancement of transportation accessibility in Yunfu, Chaozhou, Heyuan and other cities and the promotion of tourism facilities.

Third, it is necessary to understand the degree of spatial correlation and promote the coordinated development of regions. The core development area of the Guangdong–Hong Kong–Macao Greater Bay Area should be fully utilised, considering the coordinated development of other regions in Guangdong Province, which can help expand multiple core nodes, improve the traffic structure network of its surrounding and core nodes and strengthen mutual assistance and win–win results in the region and its surroundings. It is also essential to continue to develop the Metropolitan Circles of Guangzhou, Shenzhen, Pearl River West Coast, Zhan Mao and Chaoshan Jiedu and use the radiation power of megacities, including underdeveloped cities, such as Shaoguan, Qingyuan and Yunfu to closely combine core and sub-core cities to achieve integrated development.

This study comprehensively and systematically explored the spatiotemporal characteristics and evolution trends of the network structure of Guangdong's tourism economy from 2000 to 2020 using the gravity model and social network analysis method. The tourism economy of Guangdong Province is generally well developed, but individual cities differ significantly. This study can provide an empirical basis for the tourism departments of Guangdong Province and prefecture-level cities to develop policies for coordinating the development of tourism linkages. The spatial interaction theory is applied to study Guangdong Province's tourism economic network structure, which this case can further enrich.

## Author contributions

Conceptualization, Lijuan Zhang; data curation, Lijuan Zhang, Kaixiong Zhao, Zhihao Huang and Wenfu Chen; formal analysis, Lijuan Zhang, writing—original draft, Lijuan Zhang, Zhenjie Liao, Kaixiong Zhao, Zhihao Huang and Wenfu Chen; software, Lijuan Zhang; writing—review and editing, Lijuan Zhang, Azizan Marzuki and Zhenjie Liao. All authors have read and agreed to the published version of the manuscript.

## Author contribution statement

Lijuan Zhang: Conceived and designed the experiments; Performed the experiments; Analyzed and interpreted the data; Contributed reagents, materials, analysis tools or data; Wrote the paper.

Zhenjie Liao: Contributed reagents, materials, analysis tools or data; Wrote the paper.

## Funding statement

Lijuan Zhang was supported by a grant from the Guangzhou Huashang College [No.2023HSDS02 & No.2021HSXK10].

## Data availability statement

Data will be made available on request.

## Declaration of competing interest

The authors declare no conflict of interest.
